# Metabolic Syndrome and Risk of Gastrointestinal Cancers: An Investigation Using Large-scale Molecular Data

**DOI:** 10.1016/j.cgh.2021.10.016

**Published:** 2022-06

**Authors:** Joseph A. Rothwell, Mazda Jenab, Mojgan Karimi, Thérèse Truong, Yahya Mahamat-Saleh, Pietro Ferrari, S. Ghazaleh Dashti, Tilman Kühn, Amanda J. Cross, Gianluca Severi, Marc J. Gunter, Neil Murphy

**Affiliations:** ∗ Centre for Epidemiology and Population Health (U1018), Exposome and Heredity Team, Faculté de Médecine, Université Paris-Saclay, UVSQ, INSERM, Gustave Roussy, F-94805, Villejuif, France; ‡ Nutrition and Metabolism Branch, International Agency for Research on Cancer (IARC), Lyon, France; § Clinical Epidemiology and Biostatistics Unit, Murdoch Children’s Research Institute, Royal Children's Hospital, Victoria, Australia; || Institute for Global Food Security (IGFS), Queen’s University Belfast, United Kingdom; ¶ Heidelberg Institute of Global Health (HIGH), University of Heidelberg, Heidelberg, Germany; # Department of Epidemiology and Biostatistics, School of Public Health, Imperial College London, London, United Kingdom; ∗∗ Department of Statistics, Computer Science, Applications “G. Parenti,” University of Florence, Italy

**Keywords:** Cancer Genetic Risk, Cancer Prevention, Gastrointestinal Neoplasms, Molecular Epidemiology, AGM, abnormal glucose metabolism, BP, blood pressure, CI, confidence interval, HbA1c, glycated hemoglobin, HCC, hepatocellular carcinoma, HDL, high-density lipoprotein, HR, hazard ratio, IBDC, intrahepatic bile duct cancer, IDF 2005, International Diabetes Federation, 2005, MetS, metabolic syndrome, NCEP-ATPIII, National Cholesterol Education Program – Adult Treatment Panel III, PRS, polygenic risk score, UK, United Kingdom

## Abstract

**Background & Aims:**

Gastrointestinal cancer risk is influenced by the presence of metabolic syndrome (MetS). However, previous epidemiologic studies lacked full serological biomarker data for the classification of MetS, and the interaction of MetS with germline cancer risk variants is unknown.

**Methods:**

We investigated the associations between MetS and gastrointestinal cancer risk (overall, colorectal, pancreatic, esophageal adenocarcinoma, esophageal squamous cell carcinoma, stomach cardia, stomach non-cardia, hepatocellular carcinoma, and intrahepatic bile duct cancer) in 366,016 United Kingdom Biobank participants with comprehensive serum biomarker and genotype data. MetS status was determined by 3 different definitions at baseline, and, in 15,152 participants, at a repeat assessment after a median of 4.3 years of follow-up. Multivariable hazard ratios and 95% confidence intervals for cancer outcomes were estimated using Cox proportional hazards models. Analyses stratified by polygenic risk score were conducted for colorectal and pancreatic cancers.

**Results:**

During a median follow-up of 7.1 years, 4238 incident cases of a gastrointestinal cancer occurred. MetS at baseline was associated with higher risk of overall gastrointestinal cancer by any definition (hazard ratio, 1.21; 95% confidence interval, 1.13–1.29, harmonized definition). MetS was associated with increased risks of colorectal cancer, colon cancer, rectal cancer, hepatocellular carcinoma, pancreatic cancer in women, and esophageal adenocarcinoma in men. Associations for colorectal cancer and pancreatic cancer did not differ by polygenic risk score strata (*P*-heterogeneity 0.70 and 0.69, respectively), and 80% of participants with MetS at baseline retained this status at the repeat assessment.

**Conclusions:**

These findings underscore the importance of maintaining good metabolic health in reducing the burden of gastrointestinal cancers, irrespective of genetic predisposition.

## Graphical abstract

**Figure:**
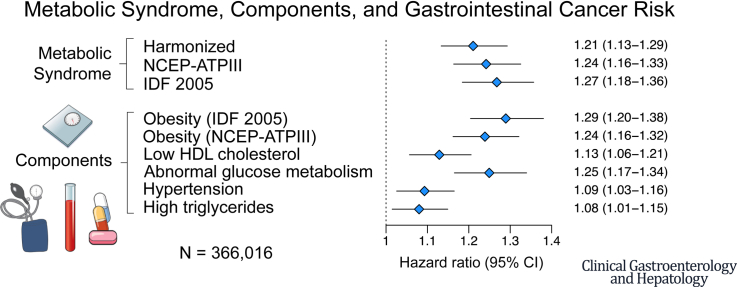

**What You Need to Know**

**Background**

Metabolic syndrome (MetS) is a cluster of metabolic abnormalities that is reported to be a risk factor for some gastrointestinal cancers. However, the use of inconsistent methods or proxies for recognized MetS definitions have limited previous cancer studies.

**Findings**

Prevalent MetS, as defined by standard molecular criteria, was associated with increased risks of colorectal cancer, colon cancer, rectal cancer, hepatocellular carcinoma, pancreatic cancer, and esophageal adenocarcinoma. For colorectal cancer and pancreatic cancer, associations did not vary across strata of polygenic risk score.

**Implications for patient care**

Given that long-term MetS status is unlikely to change in the absence of intervention, these findings highlight the importance of maintaining good metabolic health in reducing the burden of gastrointestinal cancers and developing preventative strategies.

Metabolic syndrome (MetS) refers to the simultaneous presence of several metabolic abnormalities, including abdominal obesity, abnormal glucose metabolism, elevated triglycerides, reduced high-density lipoprotein (HDL) cholesterol, and hypertension.^1^ The presence of these abnormalities promotes insulin resistance and therefore increases risk of clinical diabetes.^2^, ^3^, ^4^ MetS is a proposed risk factor for developing specific gastrointestinal cancers, including colon cancer, hepatocellular carcinoma (HCC),^3^^,^^5^, ^6^, ^7^, ^8^, ^9^, ^10^, ^11^ and pancreatic cancer in women.^3^^,^^12^ The relationships between MetS and other less common gastrointestinal cancers, such as stomach cancer and intrahepatic bile duct cancer (IBDC), are less clear as relatively few studies have been conducted to date.

Methods used to define MetS in previous cancer studies have varied, with inconsistent associations reported depending on whether recognized criteria or proxy indicators were used to replace original data (eg, using prevalent diabetes status in place of circulating glucose markers).^3^^,^^13^ Therefore, additional large-scale studies with high-quality prediagnostic epidemiologic, biomarker, and clinical data are needed to comprehensively examine the MetS and gastrointestinal cancer association. In addition, the availability of extensive genotyping data in large cohorts allows polygenic risk scores (PRS) to be derived, which may improve prediction of cancer risk at the population level,^14^ particularly where there is evidence suggesting an interaction between PRS and lifestyle or environmental risk factors. To our knowledge, the interaction between PRS and MetS and its association with cancer risk has not previously been examined.

In this study, we leverage the wealth of molecular measurements available in the United Kingdom (UK) Biobank prospective cohort to investigate the associations between MetS and risk of gastrointestinal cancers (overall, colorectal, pancreatic, esophageal adenocarcinoma and squamous cell carcinoma, stomach cardia and non-cardia, HCC, and IBDC). The availability of data on circulating concentrations of triglycerides, HDL cholesterol, and glycated hemoglobin (HbA1c) for the majority of participants at recruitment allowed us to fully adhere to the standard criteria and cut points used for MetS definitions and not rely on proxy indicators as previous studies have done. In addition, we constructed PRS for colorectal cancer and pancreatic cancer and examined the associations between MetS and these malignancies according to genetic risk strata.

## Methods

### Study Population

The UK Biobank is a large cohort of 502,656 adults initiated in 2006 that aims to investigate the genetic, lifestyle, and environmental causes of a range of diseases.^15^^,^^16^ Ethical approval was obtained from the North West Multicentre Research Ethics Committee, the National Information Governance Board for Health and Social Care in England and Wales, and the Community Health Index Advisory Group in Scotland. All participants provided written informed consent. The present study was undertaken under application number 25897. At baseline, participants completed questionnaire on socio-demographics (including age, sex, education, and Townsend deprivation score), health and medical history, lifestyle exposures (including smoking habits, dietary intakes, and alcohol consumption), early life exposures, and medication use. Physical measurements, including weight, height, and waist circumference, were taken. Systolic and diastolic blood pressure (BP) was measured from 2 separate automated readings and an average taken. Around 20,000 participants attended a repeat assessment visit between 2012 and 2013. Exclusions were made for prevalent cancer at recruitment (n = 30,296), missing MetS component data (n = 106,344), and voluntary withdrawal from the study (n = 44), leaving a final sample of 366,016 participants.

### Laboratory Methods

Blood samples (non-fasting) were collected from all participants at each assessment. Serum concentrations of triglycerides and HDL cholesterol were determined by a chemiluminescent immunoassay on a Beckman Coulter DXI 800 analyzer (Beckman Coulter, High Wycombe, UK). Coefficients of variation (CVs) of measurement ranged from 1.7% to 2.3%. HbA1c levels were determined in erythrocytes using a Variant II Turbo 2.0 high-performance liquid chromatography analyzer (Bio-Rad, Watford, UK), with CVs of 1.5% to 2.1%. Methods and quality control have previous been described.^17^ Genotyping was performed on the UKB Affymetrix Axiom array or the UK BiLEVE array^18^ with imputation using the Haplotype Reference Consortium as the main reference panel, supplemented with the UK10K and 1000 Genomes phase 3 reference panels.

### Assessment of Cancer Outcome

Incident cancer cases and cancer cases recorded first in death certificates within the UK Biobank cohort were identified through linkage to national cancer and death registries. Complete follow-up was available through March 31, 2016, for England and Wales and October 31, 2015, for Scotland. Cancer incidence data were coded using the Tenth Revision of the International Classification of Diseases. Gastrointestinal cancers included colon cancers (C18), rectal cancers (C19-20), esophageal adenocarcinomas and squamous cell carcinomas (C15), gastric cardia (C16.0) and non-cardia (C16.1-16.6) cancers, pancreatic cancers (C25), hepatocellular carcinomas (C22.0), and intrahepatic bile duct cancers (C22.1).

### Components and Definition of Metabolic Syndrome

The components of MetS are abdominal obesity, elevated circulating triglycerides, reduced circulating HDL cholesterol, abnormal glucose metabolism (AGM), and elevated BP (Supplementary Table 1). MetS status were computed based on 3 definitions: (1) the latest harmonized definition (any 3 of the components, abdominal obesity defined as per harmonized criteria),^19^ the original National Cholesterol Education Program – Adult Treatment Panel III (NCEP-ATPIII) definition which used stricter cut points for abdominal obesity,^1^ and the International Diabetes Federation (IDF) 2005 definition (abdominal obesity required, plus any 2 of the other components).^20^ Abdominal obesity was defined as waist circumference ≥102 cm or ≥88 cm (NCEP-ATPIII) or ≥94 cm and ≥80 cm (IDF 2005) in men and women, respectively. Triglycerides were considered elevated if measured at ≥1.7 mmol/L. Reduced HDL was defined as ≤1.03 mmol/L in men and ≤1.29 mmol/L in women, or regular use of cholesterol-lowering medication. AGM was defined if HbA1c ≥5.7%, regardless of diabetes status. Elevated BP was defined as ≥130 mm/Hg for systolic BP and ≥85 mm/Hg for diastolic BP, previously diagnosed high BP, or regular use of BP-lowering medication.

### Calculation of Polygenic Risk Scores for Colorectal and Pancreatic Cancer

We calculated PRS for colorectal cancer and pancreatic cancer for 363,294 (99.3%) of the eligible participants. These accounted for the majority of gastrointestinal cancers diagnosed in UK Biobank and the assembly of PRS that are strongly associated with cancer risk (hazard ratio [HR] per standard deviation increase in PRS = 1.4–1.5) has recently been described.^21^ PRS used single nucleotide polymorphisms (SNPs) that have previously been associated with colorectal cancer (n = 99) and pancreatic cancer (n = 26) at the genome-wide significance level (*P* <5 × 10^-8^).^22^^,^^23^ These were selected for independence (linkage disequilibrium r^2^ < 0.3), high imputation score, absence of allele mismatches or minor allele frequency differences >0.10 relative to the 1000 Genomes reference population, and palindromic SNPs with MAF ≥0.45. Genotypes for risk SNPs were extracted for each chromosome from imputed UK Biobank genotyping data using plink2 software, converted to dosages, and inverse variance weights applied for risk alleles. PRS for individuals were calculated as the sum of these weighted dosages. Participants were stratified into low, medium, and high PRS groups using 20th and 80th percentile cut points.

### Statistical Analysis

HRs and 95% confidence intervals (CIs) were estimated using Cox proportional hazards models. Time at entry was age at recruitment. Exit time was age at first diagnosis of incident cancer, loss to follow-up or death, or the last date at which follow-up was considered complete.

Multivariable models were adjusted for total physical activity (<10, 10–20, 20–40, 40–60, >60 MET hours/week), height (cm, continuous), alcohol consumption frequency (never, special occasions only, 1–3 times/month, 1–2 times per week, 3–4 times/week, daily or almost daily, unknown/prefer not to answer), smoking intensity (never, previous, current <15 per day, current >15 per day, current unknown intensity, unknown/prefer not to answer), frequency of red and processed meat consumption (<2 per week, 2–2.99 times/week, 3–3.99 times/week, >4/week, unknown), highest educational level (CSE/GCSE/O-level, NVQ/HND/A-level/AS-level, other professional qualification, college/university degree, missing/prefer not to answer), regular aspirin or ibuprofen use (yes/no), ever use of hormone replacement therapy (yes/no) and, where necessary, fasting time (hours, continuous). These adjustments were made for all gastrointestinal cancers, and colorectal cancer models were additionally adjusted for family history of colorectal cancer in first degree relatives (yes/no). Stratification variables were age at recruitment in 5-year categories, Townsend deprivation index quintiles, and region of the recruitment assessment center. Subgroup analyses by sex were conducted where cases >100. PRS models were additionally adjusted for genotyping array and the first 15 genetic ancestry principal components to account for population stratification. Heterogeneity across subgroups was evaluated by performing likelihood ratio tests comparing models with and without appropriate interaction terms.

Because repeat measurements of all MetS components were available for 15,152 participants, we assessed the long-term stability of MetS classifications, and additionally fit 2-way mixed models to obtain intra-class correlation coefficients as an assessment of consistency for each MetS component. Sensitivity analyses were also performed for gastrointestinal cancers overall by smoking status (never, previous, current) and excluding those participants diagnosed with a cancer within 2 years of baseline. Finally, as an additional control for bias due to different MetS durations at baseline, the analysis was performed as a nested case-control study with each case of gastrointestinal cancer matched to 5 controls on age, sex, and recruitment region, and the same adjustments as the main models.

Statistical analyses were performed using Stata 16.1 (StataCorp Inc) and R (3.6.2) statistical software. Forest plots were generated using the R package metaphor.^24^

## Results

### Baseline Characteristics of Participants

After a median follow-up of 7.1 years, 4238 incident cases of overall gastrointestinal cancers were recorded (2525 colorectal cancers, 478 pancreatic cancers, 290 esophageal adenocarcinomas, 100 esophageal squamous cell carcinomas, 111 stomach cardia cancers, 74 stomach non-cardia cancers, 112 HCC, and 108 IDBC). The prevalence of harmonized MetS among study participants was 31.9% (n = 116,624), and this group was predominantly male, of higher body mass index, lower physical activity, and higher tobacco use than participants without MetS (Table 1).

**Table 1:** Characteristics of the Study Population (n = 366,016)

	No metabolic syndrome at baseline (n = 249,392)	Prevalent metabolic syndrome (harmonized definition; n = 116,624)^a^
Gastrointestinal cancer diagnosed		
No	247,022 (99.0)	114,756 (98.4)
Yes	2370 (1.0)	1868 (1.6)
Age when attended assessment center, *years*	55.48 ± 8.15	58.40 ± 7.61
Follow-up time at cancer diagnosis, *years*	3.80 ± 2.12	3.88 ± 2.09
Participants with second assessment (of which unchanged metabolic syndrome status)		
Yes	11,036 (8635)	4116 (3275)
Sex		
Female	152,691 (61.2)	42,083 (36.1)
Male	96,701 (38.8)	74,541 (63.9)
BMI, *kg/m*^*2*^	25.9 ± 3.8	30.8 ± 4.9
Waist circumference, *cm*	85.3 ± 11.1	101.6 ± 11.3
Standing height, *cm*	167.8 ± 9.1	170.4 ± 9.4
Total physical activity level, *MET hours/week*	36.8 ± 49.5	31.6 ± 48.1
Smoking status		
Never	144,013 (57.7)	55,189 (47.3)
Previous	79,776 (32.0)	46,530 (39.9)
Current	24,548 (9.8)	14,170 (12.2)
Unknown	1055 (0.4)	735 (0.6)
Alcohol intake		
Never	9560 (3.8)	6338 (5.4)
Former	7520 (3.0)	5381 (4.6)
Current	231,765 (92.9)	104,568 (89.7)
Unknown	547 (0.2)	337 (0.3)
Socioeconomic status (Townsend deprivation index)		
Quartile 1	65,046 (26.1)	26,386 (22.7)
Quartile 4	57,996 (23.3)	33,391 (28.7)
Family history of colorectal cancer		
No	218,148 (87.5)	100,376 (86.1)
Yes	26,358 (10.6)	13,167 (11.3)
Unknown	4886 (2.0)	3081 (2.6)
Regular use of aspirin or ibuprofen		
No	190,483 (76.4)	71,242 (61.1)
Yes	56,054 (22.5)	43,521 (37.3)
Unknown	2855 (1.1)	1861 (1.6)
Red or processed meat intake, *times/week*	4.5 ± 10.3	5.6 ± 12.5
Blood pressure, *mmHg*		
Systolic	136.4 ± 19.2	146.9 ± 18.4
Diastolic	80.3 ± 10.2	86.6 ± 10.5
Glycated hemoglobin, *mmol/mol*	34.45 ± 4.03	39.71 ± 9.56
HDL cholesterol, *mmol/L*	1.56 ± 0.37	1.20 ± 0.28
Triglycerides, *mmol/L*	1.42 ± 0.74	2.45 ± 1.19
Polygenic risk score, colorectal cancer (99 SNPs)	653.3 ± 45.4	653.0 ± 45.4
Polygenic risk score, pancreatic cancer (26 SNPs)	146.0 ± 21.4	146.1 ± 21.6

Note: Data are presented as mean ± SD or number (%).

BMI, Body mass index; HDL, high-density lipoprotein; MET, metabolic equivalent of task; SD, standard deviation; SNP, single nucleotide polymorphism.

a Rate of metabolic syndrome in 353 participants excluded from the study due to prevalent gastrointestinal cancers was 44.8%, compared with 31.9% for cancer-free participants.

### Metabolic Syndrome and Risk of Gastrointestinal Cancers

#### Overall **G**astrointestinal **C**ancer

**Figure 1:**
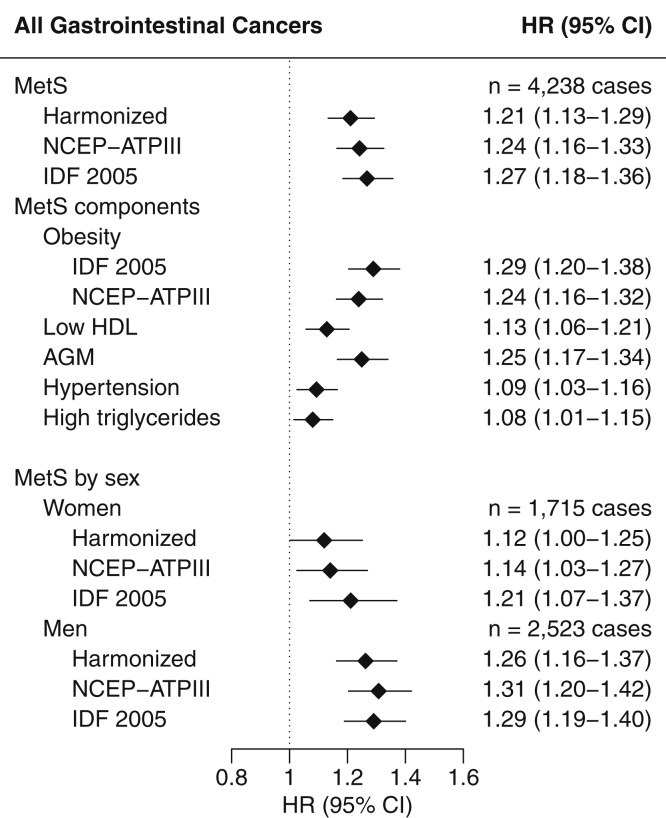
Multivariable-adjusted hazard ratios (HRs) and 95% confidence intervals (CIs) for overall gastrointestinal cancer risk and prevalent metabolic syndrome, defined by the presence of 3 or more components. AGM, Abnormal glucose metabolism; HDL, high density lipoprotein; IDF 2005, International Diabetes Federation 2005; MetS, metabolic syndrome; NCEP-ATPIII, National Cholesterol Education Program – Adult Treatment Panel III.

MetS, as classified by the harmonized definition, was associated with higher risk of developing gastrointestinal cancers (HR, 1.21 for presence versus absence of MetS; 95% CI, 1.13-1.29) (Figure 1), with similar associations for women (HR, 1.12; 95% CI, 1.00-1.25) and men (HR, 1.26; 95% CI, 1.16-1.37; *P* heterogeneity = .09). Associations were not appreciably different for the NCEP-ATPIII and IDF 2005 MetS classifications. The presence of all individual MetS components were associated with increased gastrointestinal cancer risk.

#### Colorectal Cancer

**Figure 2:**
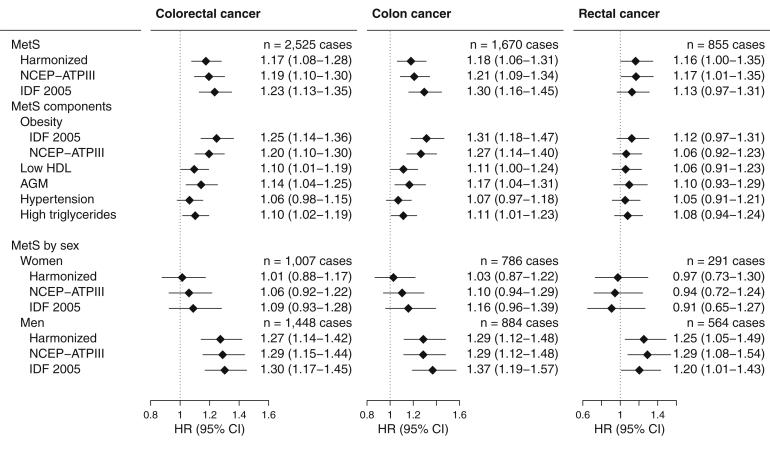
Multivariable-adjusted hazard ratios (HRs) and 95% confidence intervals (CIs) for colorectal cancer risk and prevalent metabolic syndrome, defined by the presence of 3 or more components. AGM, Abnormal glucose metabolism; HDL, high density lipoprotein; IDF 2005, International Diabetes Federation 2005; MetS, metabolic syndrome; NCEP-ATPIII, National Cholesterol Education Program – Adult Treatment Panel III.

MetS was associated with higher colorectal cancer risk (harmonized definition: HR, 1.17; 95% CI, 1.08–1.28), with a similar association observed for men (HR, 1.27; 95% CI, 1.14–1.42), but not women (HR, 1.01; 95% CI, 0.88–1.17; *P*-heterogeneity = .04). Of the individual MetS components, the presence of obesity by either definition was most strongly associated with colorectal cancer (Figure 2). Associations of similar strength were found for colon and rectal cancer (P-heterogeneity = .88, harmonized definition).

#### Esophageal Cancer and Stomach Cancer

**Figure 3:**
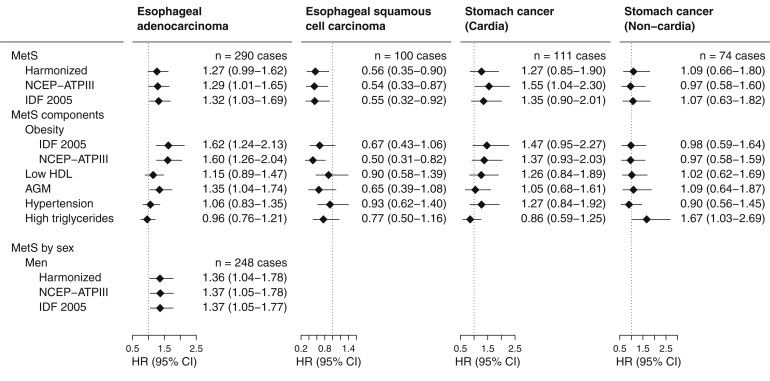
Multivariable-adjusted hazard ratios (HRs) and 95% confidence intervals (CIs) for stomach and esophageal cancer risk and prevalent metabolic syndrome, defined by the presence of 3 or more components. Sex-stratified results are only given where cases >100. AGM, Abnormal glucose metabolism; HDL, high density lipoprotein; IDF 2005, International Diabetes Federation 2005; MetS, metabolic syndrome; NCEP-ATPIII, National Cholesterol Education Program – Adult Treatment Panel III.

MetS was associated with an increased risk of esophageal adenocarcinoma by all definitions in men (85.5% of all cases; harmonized MetS: HR, 1.36; 95% CI, 1.04–1.78) (Figure 3). Of the assessed components of MetS, the presence of obesity, by either definition, was most notably associated with risk. In contrast, there was evidence for an inverse association between MetS and esophageal squamous cell carcinoma risk (HR, 0.56; 95% CI, 0.35–0.90), driven similarly by the obesity component. A positive association between MetS and cancer of the stomach cardia was found for NCEP-ATPIII MetS (HR, 1.55; 95% CI, 1.04–2.30).

#### Pancreatic Cancer, Hepatocellular Carcinoma, and Intrahepatic Bile Duct Cancers

**Figure 4:**
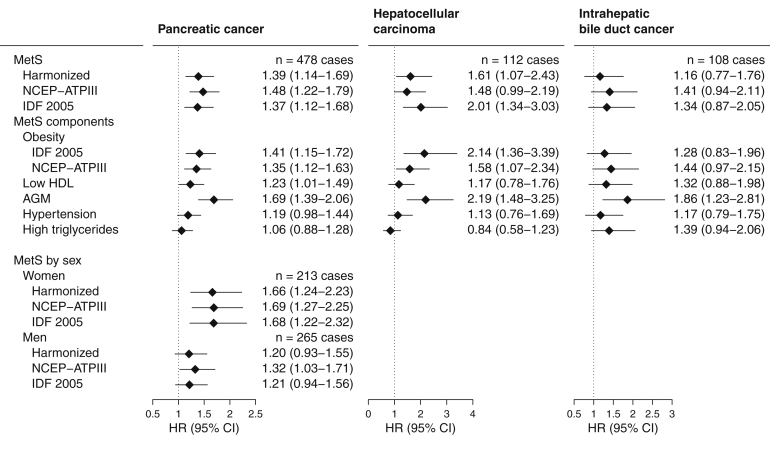
Multivariable-adjusted hazard ratios (HRs) and 95% confidence intervals (CIs) for hepatocellular carcinoma, pancreatic cancer, and bile duct cancer risk and prevalent metabolic syndrome, defined by the presence of 3 or more components. Sex-stratified results are only given where cases >100. P Heterogeneity by sex for pancreatic cancer was 0.06, 0.09, and 0.05 for harmonized, NCEP-ATPIII, and IDF 2005 MetS definitions, respectively. AGM, Abnormal glucose metabolism; HDL, high density lipoprotein; IDF 2005, International Diabetes Federation 2005; MetS, metabolic syndrome; NCEP-ATPIII, National Cholesterol Education Program – Adult Treatment Panel III.

MetS was associated with increased risk of pancreatic cancer (HR, 1.39; 95% CI, 1.14–1.69), with positive associations found particularly for obesity and AGM (Figure 4). In contrast to other cancers, associations were stronger in women than men, with a suggestion of heterogeneity for the MetS association (P-heterogeneity = .06, harmonized MetS) (see Supplementary Table 2 for MetS components by sex). Harmonized MetS was also associated with HCC risk (HR, 1.61; 95% CI, 1.07–2.43) but not IBDC risk (HR, 1.16; 95% CI, 0.77–1.76).

### Associations According to PRS Strata

**Figure 5:**
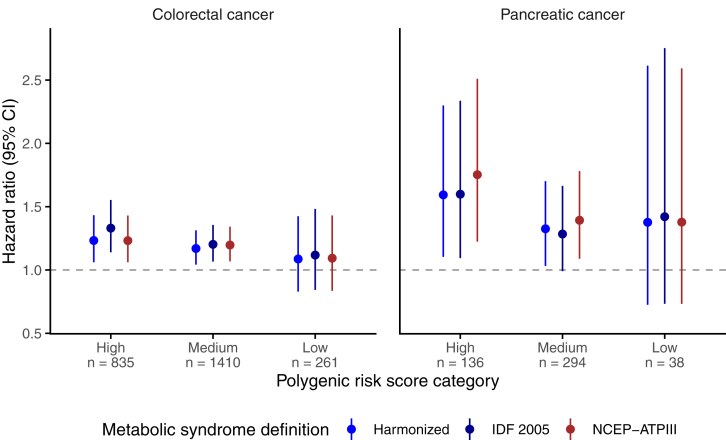
Multivariable-adjusted hazard ratios and 95% confidence intervals (CIs) for associations of prevalent metabolic syndrome with colorectal cancer and pancreatic cancer by polygenic risk score category. IDF 2005, International Diabetes Federation 2005; MetS, metabolic syndrome; NCEP-ATPIII, National Cholesterol Education Program – Adult Treatment Panel III. P Heterogeneity across PRS strata was 0.70 and 0.69 for colorectal and pancreatic cancers, respectively.

Positive associations between harmonized MetS and colorectal cancer or pancreatic cancer were generally maintained within PRS strata (eg, HR, 1.17; 95% CI, 1.04–1.31 and HR, 1.33; 95% CI, 1.03–1.70 for medium PRS categories in the 2 cancers respectively) (Figure 5) and no evidence of interaction was detected for either cancer (*P* = .70 and .69, respectively).

### MetS Stability

Of those participants with prevalent MetS were reassessed by the UK Biobank after a median of 4.3 years, 80.0%, 78.1%, and 77% retained this status by harmonized, NCEP-ATPIII, and IDF 2005 definitions, respectively (Supplementary Table 3).

### Sensitivity Analysis

Associations for gastrointestinal cancers were weakened in current smokers, although no heterogeneity was detected (*P* = .18) (Supplementary Table 4). Associations were systematically unchanged or became stronger when cases diagnosed within the first 2 years of follow-up were excluded. Intra-class correlation coefficients for 2 timepoints over a median of 4.3 years were highest for waist circumference (0.86; 95% CI, 0.85–0.86) and lowest for diastolic BP (0.62; 95% CI, 0.61–0.63) (Supplementary Table 5, Supplementary Table 6). Associations for gastrointestinal cancers were unchanged when expressed as odds ratios via an equivalent nested case-control study design (Supplementary Table 7).

## Discussion

We comprehensively examined the association between MetS and gastrointestinal cancer risk. MetS, independently of prevalent diabetes, was positively associated with overall gastrointestinal cancer risk for both men and women, as were its individual components. MetS was strongly associated with increased risks of colorectal cancer, HCC, pancreatic cancer, and esophageal adenocarcinoma in men. MetS associations with risks of colorectal and pancreatic cancers remained consistent across PRS groups, and most participants with prevalent MetS at baseline retained this status after more than 4 years of follow-up.

Chronic obesity-associated inflammation, hyperglycemia, and hyperinsulinemia are the mechanisms associated with MetS that are considered to influence gastrointestinal neoplasia.^2^^,^^25^^,^^26^ Visceral adipose tissue, for example, produces adipokines that inhibit apoptosis while promoting cell proliferation.^25^ Also, the exposure of cancer cells to high insulin levels stimulates mitogenesis.^2^ This was reflected in the strength of associations for individual obesity and AGM components. However, the large sample size and rigorous adherence to standard MetS definitions confirmed the presence of associations for the dyslipidemia and BP components. Elevated triglycerides remained associated with the risk of colorectal and colon cancer. Obesity is linked to elevated triglycerides; in visceral adiposity, energy is stored in this form.^25^

Overall, associations for colon cancer appeared to be driven by male cancers, with higher magnitude HRs observed, although there was weak evidence suggesting heterogeneity by sex. Rectal cancer followed a similar pattern, with a significant association detected for harmonized and NCEP-ATPIII definitions, unlike in the EPIC study.^11^ In contrast, MetS-pancreatic cancer associations were driven by female cancers, as reported by a Korean prospective study^12^ and a large meta-analysis on MetS and cancer.^3^ Metabolic dysregulation promotes insulin resistance, and the pancreas is exposed to high levels of endogenous insulin, which has mitogenic and anti-apoptotic effects.^2^ Although both obesity and AGM were strongly associated with pancreatic cancer risk overall, obesity was the main driver of the stronger MetS associations in women. This finding is consistent with an National Institues of Health-AARP study that reported a positive association between waist circumference and pancreatic cancer for women but not men.^27^ Additional studies are required to examine which specific aspects of central obesity-related metabolic dysregulation may be driving pancreatic cancer development and how this process may differ by sex.

Stratification of study participants by PRS has usually been implemented with the aim of optimizing colorectal cancer screening strategies. We assessed whether genetic risk category was a modifier of the association between MetS and colorectal or pancreatic cancer, and found no evidence in support. This suggests that all the population, regardless of their genetic profile, may be susceptible to the adverse tumorigenic effects of poor metabolic health, and priority for intervention in metabolic health should therefore not be based on genetic risk. To our knowledge, this is the first study to examine genetic risk of cancer in conjunction with overall metabolic dysregulation. However, a similar conclusion was reached by a German study that found no heterogeneity of the association between colorectal cancer risk and non-steroidal anti-inflammatory drug use across strata of PRS.^28^

HCC and IBDC are less prevalent than pancreatic cancer in Western populations, although the global incidence of the latter is rising.^29^ In support of a recent meta-analysis,^6^ we found MetS to be strongly associated with HCC, whereas the presence of AGM, but not MetS overall, was associated with IBDC risk. Few studies have examined MetS in relation to minor hepatobiliary cancers; a composite MetS score was found to be associated with gallbladder cancer in women in the Me-Can study,^30^ but no other data were available.

Esophageal adenocarcinoma and squamous cell carcinoma differ markedly in their patterns of incidence and etiologic factors. Adenocarcinoma risk was positively associated with MetS in men and squamous cell cancer risk inversely associated with MetS overall. Obesity exerted a disproportionate influence in these associations, consistent with findings from the large Me-Can study.^31^ As well as influencing adenocarcinoma risk through chronic inflammation, obesity is thought to increase risk separately through gastroesophageal reflux and its known progression to Barrett’s esophagus.^32^ Inverse associations of squamous cell carcinoma risk with obesity have previously been reported.^33^ Residual confounding by smoking, which is often more common in leaner study participants, has been proposed as an explanation for this finding.

The study manifests notable strengths. Firstly, high-quality baseline measurements of serum biomarkers allowed AGM and dyslipidemia to be objectively assessed, independently of diabetes. Comprehensive genotype data allowed the incorporation of PRS into the analysis. Furthermore, owing to the cohort’s repeat assessment, we have shown for the first time that MetS status is unlikely to change over a 4-year period in the absence of any intervention. Some limitations should be considered. Full MetS data were not available for around one-quarter of the cohort, and the duration of MetS at baseline was unknown. Participants did not fast prior to blood draw, potentially leading to inconsistencies in biomarker measurements. Furthermore, the relatively short follow-up limited the examination of rarer gastrointestinal cancers. Finally, generalization to other populations should be made with caution, given the “healthy participant” bias within the UK Biobank.^34^

In summary, predominantly long-term MetS was robustly associated with risk of developing overall gastrointestinal cancer, colorectal cancer, pancreatic cancer, and HCC, regardless of baseline genetic risk. Although obesity and AGM were most influential in these associations, all other components were associated with overall gastrointestinal cancer risk. Given that MetS status is unlikely to change long-term, these findings highlight the importance of maintaining good metabolic health in reducing the burden of gastrointestinal cancers and should assist in the development of preventative strategies.

## Figures and Tables

**Supplementary Table 1:** Definitions of Metabolic Syndrome as used in the Study

Metabolic syndrome definition	Harmonized (2009)	National Cholesterol Education Program – Adult Treatment Panel III (NCEP-ATPIII) (2005)	International Diabetes Federation 2005 (IDF 2005)
	Three or more of the following:	Three or more of the following:	Required:
Abdominal obesity	≥94 cm in men, ≥80 cm in women	≥102 cm in men, ≥88 cm in women	≥94 cm in men, ≥80 cm in women
			Plus any two of the following:
Elevated triglycerides	≥150 mg/dL (1.7 mmol/L)	≥150 mg/dL (1.7 mmol/L)	≥150 mg/dL (1.7 mmol/L)
Reduced HDL cholesterol	≤40 mg/dL (1.03 mmol/L) in men, ≤50 mg/dL (1.29 mmol/L) in women, or use of cholesterol-lowering medications	≤40 mg/dL (1.03 mmol/L) in men, ≤50 mg/dL (1.29 mmol/L) in women, or use of cholesterol-lowering medications	≤40 mg/dL (1.03 mmol/L) in men, ≤50 mg/dL (1.29 mmol/L) in women, or use of cholesterol-lowering medications
Abnormal glucose metabolism	Glycated hemoglobin ≥5.7% of total	Glycated hemoglobin ≥5.7% of total	Glycated hemoglobin ≥5.7% of total
Elevated blood pressure	Systolic ≥130, diastolic ≥85 mmHg, previously diagnosed hypertension, or use of anti-hypertensive medications	Systolic ≥130, diastolic ≥85 mmHg, previously diagnosed hypertension, or use of anti-hypertensive medications	Systolic ≥130, diastolic ≥85 mmHg, previously diagnosed hypertension, or use of anti-hypertensive medications

HDL, high density lipoprotein.

**Supplementary Table 2:** HRs and 95% CIs for Associations Between Metabolic Syndrome Components and Pancreatic Cancer Risk by Sex

Metabolic syndrome component	HR (95% CI)^a^^,^^b^	
	Women	Men
Obesity (IDF 2005 definition)	**1.63 (1.21–2.19)**	1.21 (0.92–1.58)
Obesity (NCEP-ATPIII definition)	**1.38 (1.04–1.83)**	**1.30 (1.01–1.69)**
HDL cholesterol	**1.45 (1.09–1.93)**	1.08 (0.83–1.41)
Abnormal glucose metabolism	**1.82 (1.35–2.44)**	**1.61 (1.23–2.09)**
Hypertension	**1.44 (1.09–1.91)**	0.99 (0.76–1.28)
High triglycerides	1.13 (0.85–1.50)	1.00 (0.78–1.28)

NOTE: Boldface indicates statistical significance.

CI, Confidence interval; HR, hazard ratio; IDF 2005, International Diabetes Federation 2005; NCEP-ATPIII, National Cholesterol Education Program – Adult Treatment Panel III.

a Multivariable models were adjusted for total physical activity (<10, 10–20, 20–40, 40–60, >60 MET hour/week), height (cm, continuous), alcohol consumption frequency (never, special occasions only, 1–3 times/month, 1–2 times per week, 3–4 times/week, daily or almost daily, unknown/prefer not to answer), smoking intensity (never, previous, current <15 per day, current ≥15 per day, current unknown intensity, unknown/prefer not to answer), frequency of red and processed meat consumption (<2 per week, 2–2.99 times/week, 3–3.99 times/week, ≥4/week, unknown), educational level (CSE/GCSE/O-level, NVQ/HND/A-level/AS-level, other professional qualification, college/university degree, missing/prefer not to answer), regular aspirin or ibuprofen use (yes/no), ever use of hormone replacement therapy (yes/no) and, where appropriate, fasting time (hours, continuous).

b HRs are given for the presence compared with the absence of each component.

**Supplementary Table 3:** Assessment of Metabolic Syndrome at Baseline and Repeat Assessment in a Subset of Participants

Metabolic syndrome status	*N* at baseline	Of which unchanged at repeat assessment^a^ n (%)
Harmonized		
Absent	11,036	8635 (78.2)
Prevalent	4116	3275 (80.0)
Total	15,152	11,910 (78.6)
NCEP-ATPIII		
Absent	11,450	9031 (78.9)
Prevalent	3702	2892 (78.1)
Total	15,152	11,923 (78.7)
IDF 2005		
Absent	11,831	9999 (84.5)
Prevalent	3321	2555 (77.0)
Total	15,152	12,554 (82.9)

IDF 2005, International Diabetes Federation 2005; NCEP-ATPIII, National Cholesterol Education Program – Adult Treatment Panel III.

a After a median duration of 4.3 years.

**Supplementary Table 4:** HRs and 95% CIs for Associations Between Metabolic Syndrome, Its Individual Components, and Gastrointestinal Cancer Risk, by Smoking Status

Metabolic syndrome definition or component	Never-smokers	HR (95% CI)^a^^,^^b^	Current smokers
		Former smokers	
N for cases	1813	1862	536
MetS (Harmonized)^c^	**1.17 (1.06–1.30)**	**1.27 (1.15–1.41)**	1.11 (0.93–1.34)
MetS (NCEP-ATPIII)	**1.18 (1.07–1.31)**	**1.30 (1.18–1.43)**	**1.24 (1.03–1.48)**
MetS (IDF 2005)	**1.24 (1.12–1.38)**	**1.32 (1.20–1.46)**	1.16 (0.97–1.40)
Obesity (IDF 2005 definition)	**1.23 (1.11–1.36)**	**1.28 (1.16–1.41)**	1.11 (0.93–1.34)
Obesity (NCEP-ATPIII definition)	**1.30 (1.18–1.44)**	**1.35 (1.22–1.51)**	1.07 (0.88–1.28)
HDL cholesterol	1.05 (0.95–1.16)	**1.22 (1.11–1.35)**	1.15 (0.96–1.39)
Abnormal glucose metabolism	1.10 (0.98–1.23)	**1.39 (1.26–1.54)**	**1.20 (1.00–1.45)**
Hypertension	**1.11 (1.01–1.22)**	1.03 (0.93–1.13)	**1.24 (1.04–1.49)**
High triglycerides	**1.10 (1.00–1.21)**	**1.09 (1.00–1.20)**	0.94 (0.79–1.12)

Note: Boldface indicates statistical significance.

CI, Confidence interval; HDL, high-density lipoprotein; HR, hazard ratio; IDF 2005, International Diabetes Federation 2005; MetS, metabolic syndrome; NCEP-ATPIII, National Cholesterol Education Program – Adult Treatment Panel III.

a Multivariable models were adjusted for total physical activity (<10, 10–20, 20–40, 40–60, >60 MET hour/week), height (cm, continuous), alcohol consumption frequency (never, special occasions only, 1–3 times/month, 1–2 times per week, 3–4 times/week, daily or almost daily, unknown/prefer not to answer), frequency of red and processed meat consumption (<2 per week, 2–2.99 times/week, 3–3.99 times/week, ≥4/week, unknown), educational level (CSE/GCSE/O-level, NVQ/HND/A-level/AS-level, other professional qualification, college/university degree, missing/prefer not to answer), regular aspirin or ibuprofen use (yes/no), ever use of hormone replacement therapy (yes/no) and, where appropriate, fasting time (hours, continuous).

b HRs are given for classified positive compared with negative for each component.

c *P* value for heterogeneity across strata of smoking status = .18.

**Supplementary Table 5:** HRs and 95% CIs for Associations Between MetS and Gastrointestinal Cancers Overall and Excluding Cases Diagnosed Within 2 Years of Baseline

	N for cases	HR and 95% CI for prevalent MetS^a^^,^^b^		
		Harmonized	NCEP-ATPIII	IDF 2005
All gastrointestinal cancers				
All	4238	**1.21 (1.13–1.29)**	**1.24 (1.16–1.33)**	**1.27 (1.18–1.36)**
<2-year cases excluded	3224	**1.25 (1.16–1.35)**	**1.27 (1.18–1.37)**	**1.33 (1.23–1.44)**
Colorectal cancer				
All participants	2,525	**1.17 (1.08–1.28)**	**1.19 (1.10–1.30)**	**1.23 (1.13–1.35)**
<2-year cases excluded	1,893	**1.20 (1.09–1.32)**	**1.20 (1.09–1.33)**	**1.28 (1.16–1.42)**
Colon cancer				
All	1,670	**1.18 (1.06–1.31)**	**1.21 (1.09–1.34)**	**1.30 (1.16–1.45)**
<2-year cases excluded	1,253	**1.19 (1.06–1.35)**	**1.20 (1.06–1.36)**	**1.32 (1.16–1.49)**
Rectal cancer				
All	855	**1.16 (1.00–1.35)**	**1.17 (1.01–1.35)**	1.13 (0.97–1.31)
<2-year cases excluded	640	**1.21 (1.02–1.43)**	**1.20 (1.01–1.42)**	**1.22 (1.03–1.46)**
Esophageal adenocarcinoma				
All	248	1.27 (0.99–1.62)	**1.29 (1.01–1.65)**	**1.32 (1.03–1.69)**
<2-year cases excluded	184	**1.38 (1.04–1.83)**	**1.35 (1.02–1.79)**	**1.44 (1.08–1.91)**
Esophageal squamous cell carcinoma				
All	100	0.56 (0.35–0.90)	0.54 (0.33–0.87)	0.55 (0.32–0.92)
<2-year cases excluded	77	0.54 (0.31–0.93)	0.54 (0.31–0.94)	0.54 (0.30–0.98)
Pancreatic cancer				
All	478	**1.39 (1.14–1.69)**	**1.48 (1.22–1.79)**	**1.37 (1.12–1.68)**
<2-year cases excluded	379	**1.35 (1.08–1.67)**	**1.49 (1.20–1.84)**	**1.29 (1.03–1.62)**
Hepatocellular carcinoma				
All	112	**1.61 (1.07–2.43)**	1.48 (0.99–2.19)	**2.01 (1.34–3.03)**
<2-year cases excluded	100	**1.90 (1.22–2.97)**	**1.63 (1.07–2.48)**	**2.34 (1.51–3.64)**
Stomach cancer, cardia				
All	111	1.27 (0.85–1.90)	**1.55 (1.04–2.30)**	1.35 (0.90–2.01)
<2-year cases excluded	81	1.20 (0.75–1.91)	1.46 (0.92–2.32)	1.42 (0.89–2.26)
Stomach cancer, non-cardia				
All	74	1.09 (0.66–1.80)	0.97 (0.58–1.60)	1.07 (0.63–1.82)
<2-year cases excluded	59	1.09 (0.62–1.91)	0.97 (0.55–1.71)	1.15 (0.64–2.07)
Intrahepatic bile duct cancer				
All	108	1.16 (0.77–1.76)	1.41 (0.94–2.11)	1.34 (0.87–2.05)
<2-year cases excluded	77	1.19 (0.73–1.94)	1.49 (0.93–2.40)	1.43 (0.86–2.35)

Note: Boldface indicates statistical significance.

AGM, Abnormal glucose metabolism; CI, confidence interval; HDL, high density lipoprotein; HR, hazard ratio; IDF 2005, International Diabetes Federation 2005; MetS, metabolic syndrome; NCEP-ATPIII, National Cholesterol Education Program – Adult Treatment Panel III.

a Multivariable models were adjusted for total physical activity (<10, 10–20, 20–40, 40–60, >60 MET hour/week), height (cm, continuous), alcohol consumption frequency (never, special occasions only, 1–3 times/month, 1–2 times per week, 3–4 times/week, daily or almost daily, unknown/prefer not to answer), smoking intensity (never, previous, current <15 per day, current ≥15 per day, current unknown intensity, unknown/prefer not to answer), frequency of red and processed meat consumption (<2 per week, 2–2.99 times/week, 3–3.99 times/week, ≥4/week, unknown), educational level (CSE/GCSE/O-level, NVQ/HND/A-level/AS-level, other professional qualification, college/university degree, missing/prefer not to answer), regular aspirin or ibuprofen use (yes/no), ever use of hormone replacement therapy (yes/no), family history of colorectal cancer in first degree relatives (yes/no) and, where appropriate, fasting time (hours, continuous).

b HRs are given for MetS detected versus not detected.

**Supplementary Table 6:** Intra-class Correlation Coefficients for Metabolic Syndrome Components

	Number of time points	N for subset	Intra-class correlation coefficient (95% CI)^a^
Waist circumference			
All	3	7061	0.86 (0.85–0.86)
Male		3569	0.81 (0.80–0.82)
Female		3492	0.81 (0.08–0.82)
Triglycerides			
All	2	15775	0.64 (0.63–0.65)
Male		8011	0.61 (0.59–0.62)
Female		7764	0.66 (0.65–0.68)
HDL cholesterol			
All	2	12790	0.85 (0.84–0.85)
Male		6627	0.81 (0.81–0.82)
Female		6163	0.81 (0.80–0.82)
Glycated hemoglobin			
All	2	12240	0.78 (0.77–0.79)
Male		6058	0.80 (0.79–0.81)
Female		6182	0.76 (0.75–0.77)
Blood pressure, diastolic			
All	2	18387	0.62 (0.61–0.63)
Male		9090	0.57 (0.56–0.59)
Female		9297	0.64 (0.63–0.65)
Blood pressure, systolic			
All	2	18383	0.66 (0.65–0.67)
Male		9088	0.62 (0.61–0.63)
Female		9295	0.69 (0.68–0.70)

CI, confidence interval; HDL, high density lipoprotein.

a Calculated from 2-way linear mixed effects models in a subset of around 20,000 participants who were reassessed after a median of 4.3 years after baseline.

**Supplementary Table 7:** ORs and 95% CIs for Associations Between Metabolic Syndrome, Its Individual Components, and Gastrointestinal Cancer Risk, by Smoking Status

Metabolic syndrome definition or component	OR (95% CI)^a^^,^^b^	HR (95% CI)^a^^,^^b^
N for study	4238 cases + 21,190 controls	366,016
All participants		
MetS (Harmonized)	**1.23 (1.15–1.32)**	**1.21 (1.13–1.29)**
MetS (NCEP-ATPIII)	**1.26 (1.17–1.35)**	**1.24 (1.16–1.33)**
MetS (IDF 2005)	**1.29 (1.20–1.39)**	**1.27 (1.18–1.36)**
Women		
MetS (Harmonized)	1.12 (0.99–1.27)	**1.12 (1.00–1.25)**
MetS (NCEP-ATPIII)	**1.15 (1.02–1.29)**	**1.14 (1.03–1.27)**
MetS (IDF 2005)	**1.20 (1.05–1.38)**	**1.21 (1.07–1.37)**
Men		
MetS (Harmonized)	**1.30 (1.19–1.42)**	**1.26 (1.16–1.37)**
MetS (NCEP-ATPIII)	**1.34 (1.22–1.46)**	**1.31 (1.20–1.42)**
MetS (IDF 2005)	**1.33 (1.22–1.46)**	**1.29 (1.19–1.40)**
Individual components		
Obesity (IDF 2005 definition)	**1.26 (1.17–1.35)**	**1.24 (1.16–1.32)**
Obesity (NCEP-ATPIII definition)	**1.32 (1.23–1.42)**	**1.29 (1.20–1.38)**
HDL cholesterol	**1.14 (1.07–1.23)**	**1.13 (1.06–1.21)**
Abnormal glucose metabolism	**1.29 (1.19–1.39)**	**1.25 (1.17–1.34)**
Hypertension	**1.08 (1.01–1.16)**	**1.09 (1.03–1.16)**
High triglycerides	**1.08 (1.01–1.16)**	**1.08 (1.01–1.15)**

Note: Boldface indicates statistical significance.

CI, Confidence interval; HR, hazard ratio; IDF 2005, International Diabetes Federation 2005; NCEP-ATPIII, National Cholesterol Education Program – Adult Treatment Panel III; OR, odds ratio.

a Multivariable models were adjusted for total physical activity (<10, 10–20, 20–40, 40–60, >60 MET hour/week), height (cm, continuous), alcohol consumption frequency (never, special occasions only, 1–3 times/month, 1–2 times per week, 3–4 times/week, daily or almost daily, unknown/prefer not to answer), smoking intensity (never, previous, current <15 per day, current ≥15 per day, current unknown intensity, unknown/prefer not to answer), frequency of red and processed meat consumption (<2 per week, 2–2.99 times/week, 3–3.99 times/week, ≥4/week, unknown), educational level (CSE/GCSE/O-level, NVQ/HND/A-level/AS-level, other professional qualification, college/university degree, missing/prefer not to answer), regular aspirin or ibuprofen use (yes/no), ever use of hormone replacement therapy (yes/no) and, where appropriate, fasting time (hours, continuous).

b ORs and HRs and are given for classified positive compared with negative for each component.
